# SB772077B, A New Rho Kinase Inhibitor Enhances Aqueous Humour Outflow Facility in Human Eyes

**DOI:** 10.1038/s41598-018-33932-8

**Published:** 2018-10-19

**Authors:** Soundararajan Ashwinbalaji, Srinivasan Senthilkumari, Chidambaranathan Gowripriya, Subbaiah Krishnadas, B’ Ann T. Gabelt, Paul L. Kaufman, Veerappan Muthukkaruppan

**Affiliations:** 1 Department of Ocular Pharmacology, Aravind Medical Research Foundation, #1, Anna Nagar, Madurai-20, Tamilnadu India; 2 Department of Immunology & Stem Cell Biology, Aravind Medical Research Foundation, #1, Anna Nagar, Madurai-20, Tamilnadu India; 3 Glaucoma Clinic, Aravind Eye Hospital, #1, Anna Nagar, Madurai-20, Tamilnadu India; 4 Department of Ophthalmology & Visual Sciences, University of Wisconsin, Madison, Wisconsin USA; 5 Advisor, Aravind Medical Research Foundation, #1, Anna Nagar, Madurai-20, Tamilnadu India

## Abstract

We investigated the effect of a new Rho kinase inhibitor, SB772077B (SB77) on aqueous outflow facility (OF) in human eyes using human organ-cultured anterior segment (HOCAS). IOP was monitored for 24 h post-treatment with either SB77 (0.1/10/50 µM) or vehicle after a stable baseline pressure. The hydrodynamic pattern of aqueous outflow was analysed by labelling outflow pathway with red fluorescent microspheres. The effect of SB77 on cell morphology, actin stress fibers, focal adhesions, ECM, status of RhoA activation and myosin light chain phosphorylation (p-MLC) were evaluated and compared with Y27632, by immunostaining using primary human trabecular meshwork (HTM) cells. Following 24 h treatment, SB77 increased OF by 16% at 0.1 µM (N = 6), 29% at 10 µM (N = 8; p = 0.018) and 39% at 50 µM (N = 8; p = 0.004) in human eyes. There was an overall increase in tracer quantity and in area along inner wall of Schlemm’s canal. Treatment with SB77 showed no evidence of cytotoxicity and caused a significant reduction in the expression of fibrotic markers compared to Y27632. The present findings indicate that SB77 treatment was effective in enhancing OF and reducing fibrotic markers in an *ex vivo* model. Thus SB77 may be a potential clinical candidate for the management of glaucoma.

## Introduction

Glaucoma is the leading cause of irreversible blindness in the world, with a prevalence of 3.54% in the 40–80 year old population^1^. Primary open angle glaucoma (POAG) is the most predominant form of glaucoma, with 57.5 million people affected globally and their numbers predicted to increase to 65.5 million in 2020^2,3^. POAG is characterized by progressive retinal ganglion cell loss, optic nerve damage and visual field loss leading to blindness^2^.

A major causal risk factor for POAG is elevated intraocular pressure (IOP) caused by increased resistance to aqueous humour (AH) outflow localized within the conventional/trabecular meshwork (TM) pathway^4^. The increased outflow resistance occurs mainly in the juxtacanalicular TM (JCT), the portion closest to Schlemm’s canal (SC), and in the inner wall endothelial lining of SC^4^. Regulation of conventional outflow resistance is dynamic and likely involves multiple signalling molecules including bioactive lipids, cytokines, nucleotides and gases^5^. Accumulating evidence suggests that actin cytoskeleton–modulating signals are involved in aqueous outflow regulation^6^.

Rho is a small GTPase that is involved in the regulation of many cell processes including contraction, cytoskeleton organization, adhesive interactions, trafficking and permeability. Activation of the RhoA-ROCK pathway has been demonstrated to decrease AH outflow through the TM by inducing alterations in cell contraction, actomyosin assembly, cell adhesion and extracellular matrix (ECM) synthesis^5,7^. Primary molecules that transmit RhoA-ROCK signalling (e.g.: myosin light chain phosphatase, LIM kinase, cofilin) are reported to be expressed in human TM with mediators for this signalling existing in AH^6–9^. Inhibition of this pathway is an attractive strategy to increase OF for the management of glaucoma. Rho kinase inhibitors (RKIs) lower IOP in animal models and humans in association with decreased myosin II phosphorylation and disruption of actin stress fibres^10,11^. SB77 is a novel aminofuran-based RKI with anti-inflammatory activity^12^. It is reported to decrease pulmonary and systemic blood pressure and exhibits vasodialatory activity that is more potent than Y27632 and fasudil^12,13^. The IOP lowering property of the SB77 has not been reported. Therefore, in the present study, the IOP lowering property of SB77 was analysed *ex vivo* in human organ-cultured anterior segment (HOCAS). In addition, the associated effect of SB77 on fibrotic markers was investigated using primary cultures of human TM cells.

## Results

The mean (±SD) donor age was 71.5 ± 15.2 years and anterior segments were cultured within 30.5 h of enucleation (mean elapsed time between enucleation and culture was 22.8 ± 7.7 h except 3 eyes which got longer culture time of 49.3 ± 4.7 h. The overall culture time for the studied eye was 26.4 ± 11.8 h (Table 1).

**Table 1:** Characteristics of Human donor Eyes used for the Experiment.

SB77 Treatment (µM)	Code	Age	Sex	Cause of Death	Time between Death and Enucleation (h)	Time between Enucleation and Culture (h)
0.1	1	86	F	Respiratory Arrest	4.5	25
	2	80	F	Heart attack	1.5	8
	3	77	F	Renal Failure	1.25	29
	4	72	M	Myocardial infarction	3.5	24.5
	5	78	F	Cardiac arrest	1.5	12
	6	81	F	Cerebrovascular accident	3	17
10	7	79	M	Myocardial infarction	4.5	14
	8	40	F	Myocardial infarction	3	28
	9	78	M	Natural	4.75	25
	10	60	M	Natural	4	51
	11	72	M	Cardiac Arrest	2.5	20
	12	70	F	Cardiac Arrest	1	14.5
	13	75	F	Respiratory arrest	2	34
	14	81	F	Respiratory arrest	1	33
50	15	80	F	Natural	3.25	36
	16	30	M	Respiratory arrest	3.5	24
	17	75	F	Natural	2.5	44
	18	42	M	Heart attack	4.5	53
	19	75	M	Respiratory arrest	2	18
	20	80	M	Cardiac Arrest	2.75	25
	21	89	M	Respiratory illness	4	28
	22	74	M	Cardiac arrest	2.75	19

The mean (±SD) donor age was 71.5 ± 15.2 years and cultured within 30.5 h (22.8 ± 7.7 h) except 3 eyes (Sl. No. 10, 17 and 18; range: 44–53 h). The overall mean culture time for the studied eyes was 26.4 ± 11.8 h (38.2 h).

### Effect of SB77 on OF

**Figure 1:**
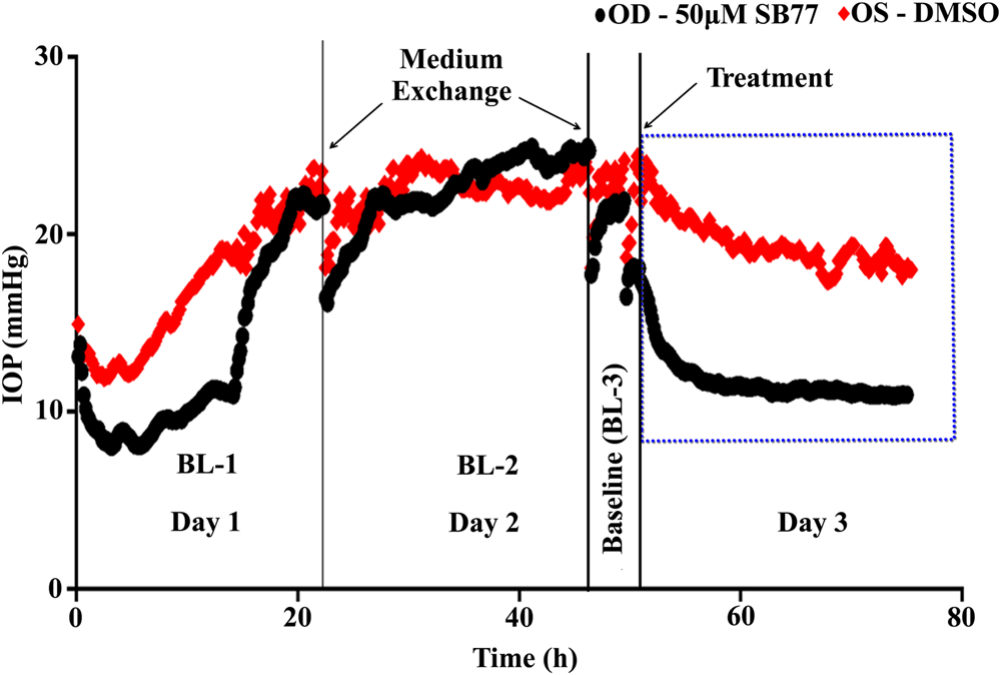
A representative intraocular pressure (IOP) graph of the anterior segment receiving SB77 treatment. The presence of SB77 decreases IOP as compared to its vehicle treated anterior segment.

The IOP lowering property of SB77 in HOCAS was investigated with three different dose levels: 0.1, 10 and 50 µM. A representative IOP profile after SB77 treatment is shown in Fig. 1. SB77 treatment induced a dose-dependent increase in OF. Following 24 h treatment, OF was increased by 16% at 0.1 µM (N = 6), 29% at 10 µM (N = 8; p = 0.018) and 39% at 50 µM (N = 8; p = 0.004) compared to vehicle control (Table 2).

**Table 2:** Effect of Lat B and SB77 on Aqueous OF in Human Eyes.

SB77 (µM)	n	Eye Pair	Baseline OF (μl/min/mmHg)	Rx-3 h/BL	Rx-12 h/BL	Rx-24 h/BL	%Change in OF			
							Baseline	3 h	12 h	24 h
0.1	6	Con	0.22 ± 0.04	1.03 ± 0.03	1.04 ± 0.02	1.02 ± 0.02	1.56 ± 23.6	5.12 ± 4.12	15.63 ± 5.59	15.9 ± 5.4
	6	Exp	0.27 ± 0.07	1.08 ± 0.04	1.20 ± 0.04	1.18 ± 0.05				
10	8	Con	0.29 ± 0.04	1.14 ± 0.05	1.12 ± 0.07	1.09 ± 0.05	−4.74 ± 12.9	4.58 ± 9.9 (p = 0.031)	27* ± 2.1 (p = 0.015)	29.34* ± 5.88 (p = 0.018)
	8	Exp	0.22 ± 0.02	1.19 ± 0.18 (p = 0.049)	1.25* ± 0.07 (p = 0.014)	1.29* ± 0.09 (p = 0.017)				
50	8	Con	0.20 ± 0.04	1.05 ± 0.04	1.15 ± 0.07	1.18 ± 0.13	−9.65 ± 11.67	21.36* ± 3.72 (p = 0.027)	28.55** ± 5.4 (p = 0.009)	39.48** ± 2.86 (p = 0.004)
	8	Exp	0.15 ± 0.02	1.28* ± 0.06 (p = 0.032)	1.47** ± 0.09 (p = 0.002)	1.64** ± 0.18 (p = 0.002)				

Outflow facility (OF) (μl per minute/mm Hg) was calculated as the ratio between the inflow rate (μl/minute) and the measured IOP (mm Hg) and the values.

are expressed as Mean ± SEM.

% Change in OF was calculated using the formula: % Change in OF = [(Treated/BL/Control/BL) −1) × 100].

Exp – Experiment; Con = Control.

*p < 0.05, **p < 0.005, (Paired-t- test).

### Hydrodynamic Flow Pattern

**Figure 2:**
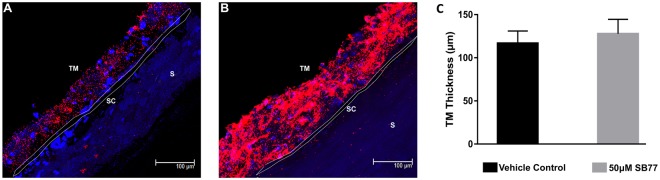
Tracer-decorated human aqueous outflow pattern. Frontal sections of anterior segments showing tracer distribution in (**A**) vehicle control, (**B**) SB77 (50 µM) treated eyes. Tracer distribution was more uniform, intense and widely spread all over the meshwork and also in the inner wall of SC in SB77-treated eyes as compared to vehicle-treated eyes. (**C**) Measurement of TM thickness in tracer decorated region. A marginal 11.2% increase in TM thickness was observed in SB77-treated eyes as compared to vehicle-treated eyes (SB77-treated eyes: 129.29 ± 14.3 µm, vehicle control eyes: 116.09 ± 16.7 µm; n = 3; p = 0.354; unpaired t-test). TM-Trabecular meshwork; SC-Schlemm’s canal and S**-**Sclera.

The fluorescent tracers were distributed segmentally in all SB77 and vehicle treated eyes. Additionally, the SB77 (50 µM) treated eyes showed an overall increase in tracer quantity and area along the inner wall of SC after 24 h treatment as compared to vehicle treated eyes (Fig. 2). The increase in tracer quantity of SB77 treated eyes was not clearly associated with TM thickness (p = 0.354).

### Effect of SB77 on Cell Viability

**Figure 3:**
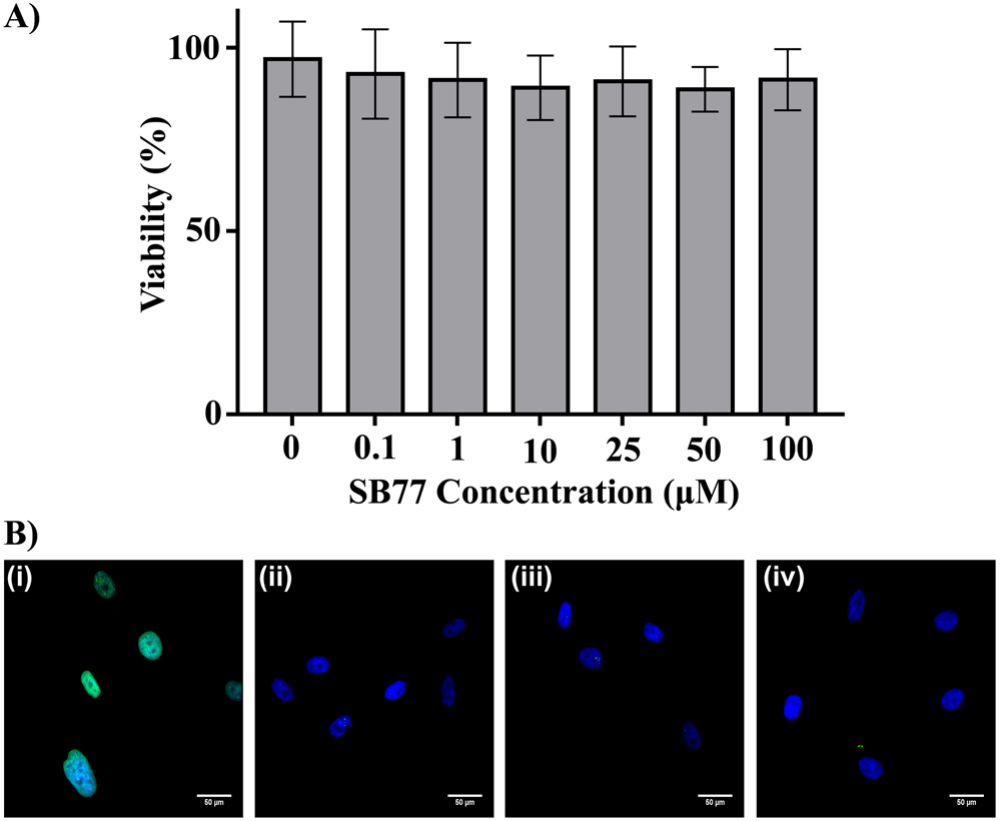
Effect of SB77 on HTM cell viability by MTT assay. (**A**) HTM cells treated with SB77 showed more than 85% (Mean percentage viability: 87.8 ± 5.9) cell viability at the studied concentrations (0.1–100 µM). The values are represented as mean ± SD. Experiments were conducted in triplicate. (**B**) Terminal deoxyribonucleotidyl transferase (TdT) - mediated fluroscein-16-dUTP nick end-labelling (TUNEL) staining on HTM cells after treatment with DNAse I (i), vehicle control (ii), SB77 (iii) and Y27632 (iv) for 24 h. TUNEL positive cells (green) were detected only in HTM cells treated with DNase I (i) (Positive control). No TUNEL positive cells were detected in other treatment groups. Cell nuclei were stained with DAPI (blue). A detailed protocol for MTT and TUNEL assay was given in method section.

Treatment with SB77 showed no significant effect on TM cell viability at all studied concentrations (Fig. 3A) (MTT assay). In TUNEL assay, both SB77 and Y27632 (50 µM) treated cells showed no evidence of apoptosis (TUNEL-positive cells) as compared to DNase I treated cells (positive control) (Fig. 3B).

### Effect of SB77 on Cell Morphology, Cytoskeleton, Focal Adhesion and ECM

**Figure 4:**
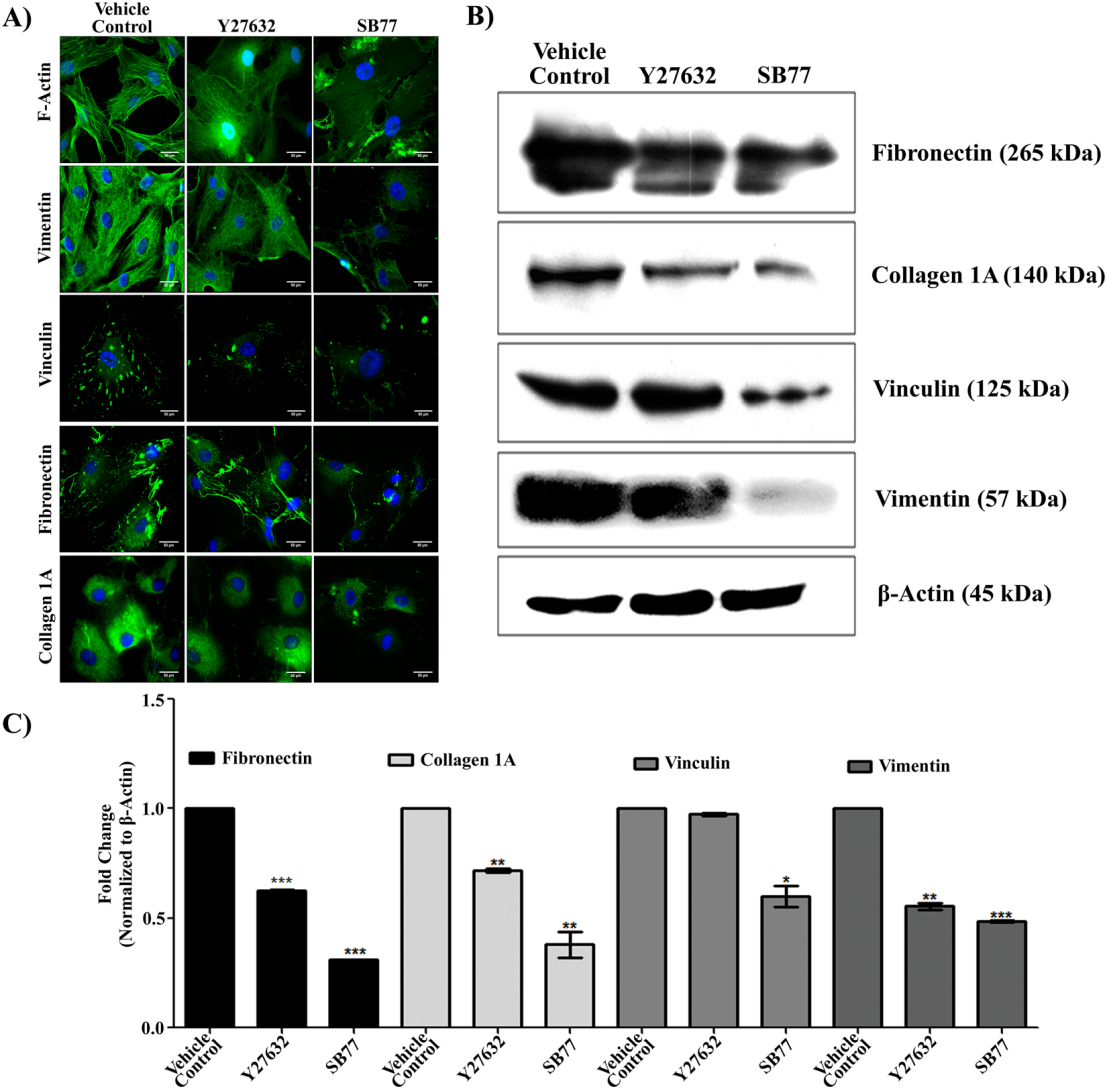
Effect of SB77 on TM actin cytoskeleton, focal adhesion (vimentin) and ECM (fibronectin). (**A**) Treatment with SB77 caused decreased positive staining of stress fibers, vimentin, vinculin, fibronectin and collagen 1 A staining. HTM cells treated with Y27632 showed similar findings; however, the observed effect was more prominent in SB77-treated cells as compared to Y27632-treated cells. The images are representative of three independent experiments. Green fluorescence indicates positivity for F-actin, vimentin, vinculin, fibronectin and collagen 1 A. Cell nuclei were stained with DAPI (blue). (**B**) Immunoblot analysis showing the effect of SB77 on the expression of focal adhesion and ECM protein. Decreased expression of vinculin, vimentin and ECM proteins was observed in HTM cells treated with both inhibitors. Treatment with SB77 showed a marked decrease as compared to vehicle control and Y27632-treated cells. The cropped images are used in the figure, and full length blots for the same are presented in S. Fig. 3. (**C**) Densitometry analysis of the results of 4B. Experiments were conducted in triplicate. Data are expressed as mean ± SD. *p < 0.05; **p < 0.001; ***p < 0.0001, paired Student’s t-test.

The morphology of HTM cells was examined after treatment with 50 µM SB77 or Y27632 for 2 h. SB77 treatment induced retraction and rounding of cells that led to stellate appearance in phase contrast images (S. Fig. 2). Immunofluorescence analysis revealed reduced actin bundles (F-actin), reduced vinculin-containing focal adhesions and reduced vimentin-containing intermediate filaments in drug treated cells as compared to vehicle controls (Fig. 4A). The observed changes were more prominent in SB77 treated cells as compared to Y27632 cells at the studied concentrations.

Immunoblot analysis revealed that HTM cells after treatment with either 50 µM SB77 or Y27632 showed significant reduction in the expression of vinculin, fibronectin, collagen 1 A (ECM protein) and vimentin as compared to vehicle controls (Fig. 4B). Densitometry analysis showed a significant reduction in fold change in SB77-treated cells compared to Y27632 treated cells (p = 0.018 (vinculin); p = 0.000 (Fibronectin); p = 0.032 (COL1A) and p = 0.076 (vimentin)) (Fig. 4C).

### Effect of SB77 on Activation of RhoA and p_MLC Phosphorylation

**Figure 5:**
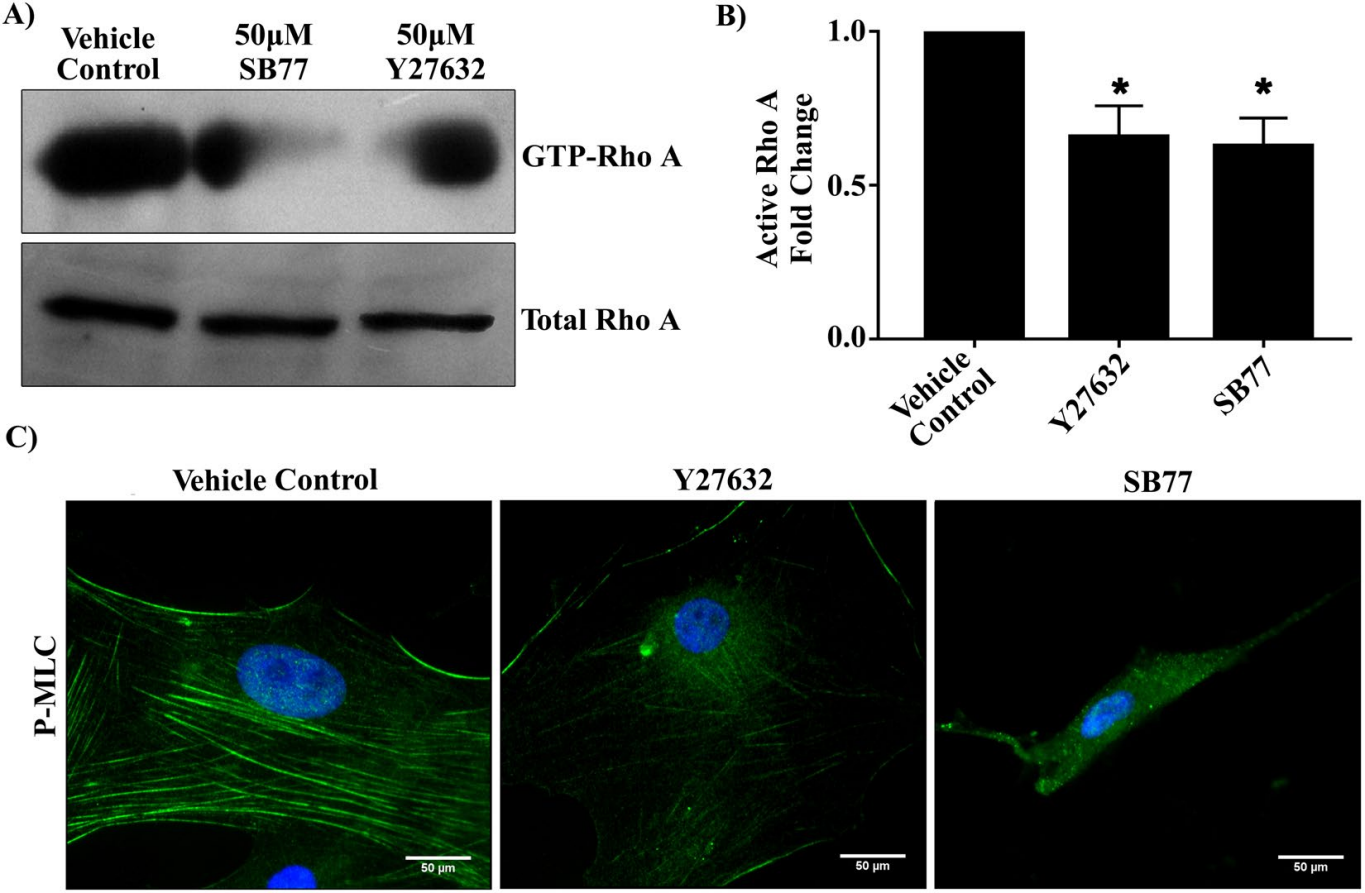
Effect of SB77 or Y27632 on activation of RhoA. (**A**) Both SB77 and Y27632 at 50 µM dose inhibited the activation of RhoA in HTM cells. The amount of reduction in activated RhoA was determined by a pull-down assay. Data shown in upper and lower panels were results of an immunoblot analysis against GTP-binding Rho (GTP-Rho) and total RhoA. The cropped blots are used in the figure and full length blots for the total RhoA and active RhoA are given in S. Fig. 4. (**B**) Densitometry of Western blots of active RhoA normalized to total RhoA. Both Rho kinase inhibitors significantly inhibited the activation of RhoA. Total RhoA was unaffected by the treatment. Data are expressed as mean ± SD. *p < 0.05 (Treated vs Control); paired Student’s t-test. (**C**) Representative immunofluorescence analysis showing reduced staining of p-MLC (green) after treatment with SB77. The observed effect was very prominent as compared to Y27632 and vehicle control groups. Cell nuclei are stained with DAPI (blue).

In order to elucidate the effect of SB77 on RhoA/ROCK signal transduction, the pull-down assay for the active form of RhoA (GTP-RhoA) was carried out in HTM cell lysates after treatment. Rho kinase inhibitor treatment significantly reduced the levels of GTP bound RhoA, thus inhibiting the activation of RhoA (p = 0.016 (vehicle control *vs* SB77-treated cells); p = 0.029 (vehicle control *vs* Y27632-treated cells)) (Fig. 5). This indicates that the enhanced OF caused by SB77 treatment is due to the inactivation of RhoA and its downstream effector p-MLC.

### Effect of SB77 on Expression of Fibrotic Markers

**Figure 6:**
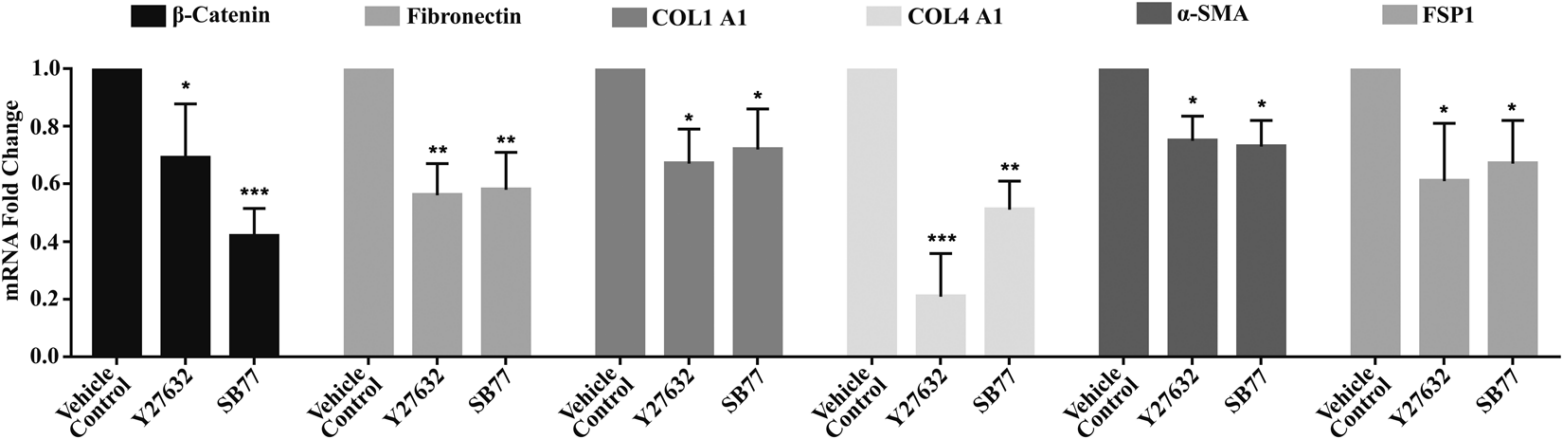
Effect of SB77 on mRNA expression of fibrotic markers by quantitative RT-PCR. A significant reduction in mRNA levels of Fibronectin (p = 0.005), COL1A1 (p = 0.015), α-SMA (p = 0.038) and FSP-1(p = 0.019) compared to vehicle control was achieved with SB77 treatment. Y27632 treatment also showed similar fold changes, except for the larger change in COL4A1. β-catenin mRNA levels were reduced significantly in SB77- treated cells as compared to vehicle controls (p = 0.000) but it was not found to be significant when compared with Y27632-treated cells (p = 0.088), whereas COL4A1 was significantly reduced in Y27632 treated cells as compared to vehicle-treated cells (p = 0.001). The fold change in gene expression was calculated by the 2^−ΔΔCt^ method, with GAPDH as a house-keeping gene. *p < 0.05; **p < 0.001; ***p < 0.0001(n = 3); Treated (SB77 or Y27632) *vs* vehicle control group, paired Student’s t-test.

A significant reduction in mRNA levels of Fibronectin (p = 0.005), COL1A1 (p = 0.015), α-SMA (p = 0.038) and FSP-1(p = 0.019) compared to vehicle control was achieved with SB77 treatment. Y27632 treatment also showed similar fold changes, except for the larger change in COL4A1. β-catenin mRNA levels were reduced significantly in SB77- treated cells as compared to vehicle controls (p = 0.000) but it was not found to be significant when compared with Y27632-treated cells (p = 0.088), whereas COL4A1 was significantly reduced in Y27632 treated cells as compared to vehicle-treated cells (p = 0.001) (Fig. 6).

## Discussion

In POAG, the impairment in OF through the TM is due to changes in cytoskeletal organization, increased tissue stiffness, increased expression and deposition of ECM proteins and elevated levels of specific cytokines and growth factors^14–18^. Elevated levels of TGFß2 are observed in AH of patients with POAG, and the TGFß2 family member Bone Morphogenesis Protein 4 has been hypothesized to be involved in the pathogenesis of POAG^19^ Recent evidence strongly suggests the involvement of Rho GTPase/ROCK signaling in the modulation of the cytoskeletal integrity of cells, synthesis of ECM components in the AH outflow tissue and the permeability of SC endothelial cells^20^.

ROCK, a member of the serine/threonine kinase family, is an important downstream effector of the small GTP-binding protein RhoA^21^. ROCK1 and ROCK2 are the two isoforms of ROCK that are reported to be ubiquitously expressed in invertebrates and vertebrates. ROCK1 is reported to be mainly expressed in circulating inflammatory cells and ROCK2 in vascular cells^22^. In human eyes, ROCK1, ROCK2 and RhoA are expressed in TM, ciliary muscle and optic nerve head^9^. Previous studies demonstrated that inhibition of the ROCK pathway decreased AH outflow resistance in perfused eyes of animals and humans, supporting its potential use in glaucoma therapy^8,11,23–25^.

The present study investigated the effect of SB77, a novel amino-furan rho kinase inhibitor, on AH OF at three dose levels in human eyes. These dose levels were chosen based on the reported IC_50_ value^12^ and also based on the toxicity profile studied in HTM cells by MTT and TUNEL assays (Fig. 4). SB77 is reported to have a high selectivity against human recombinant ROCK isoforms with an IC_50_ value of 5.6 nM as compared to other commonly used ROCK inhibitors with low selectivity (IC_50_ = 150 nM for Y27632 and 300 nM for fasudil)^12^, and is comparable to netarsudil (IC_50_ = 1 nM). Cytotoxicity assays indicate that SB77 at concentrations between 0.1–100 µM was not toxic to HTM cells (more than 95% viability; Fig. 4). Therefore, three dose levels − 0.1 µM, 10 µM, and 50 µM (18, 1800, and 9000 fold respectively vs IC_50_) - were selected for the present study.

SB77 induced dose-dependent increases in OF. SB77 at 50 µM caused a 21% increase in OF compared to baseline within 3 h (p = 0.027) and 39% at 24 h (p = 0.004) (Table 2) as compared to its baseline-corrected vehicle control. Y27632 (50 µM) induced a 60.6 ± 16.9% increase in OF after 3 h of perfusion in human eyes^26^, which is approximately 2.8 fold higher than the corresponding SB77-induced OF increase observed in the present study. This could be due either to Y27632 being more effective in enhancing OF than SB77 or to the different perfusion techniques. The present study utilized an isoated anterior segment perfusion culture system, whereas in the Yang *et al*. study a whole eye perfusion technique was used. We observed that SB77 required more time than Y27632 to enhance OF. The maximum response to 50 µM SB77 treatment had not reached a plateau at 24 h (Fig. 2), perhaps because the drug response is greatly influenced by the freshness of the tissue^27^. In our present study, the overall mean (SD) elapsed time between enucleation and culture was 38.2 h (26.4 ± 11.8 h) whereas in the study by Yang *et al*., the mean post-mortem time to culture was 22 h^26^. Also, there may be differences in responsiveness of ethnic groups to SB77 (Indian eyes used in the current study versus the American eyes used in Yang’s study) or differences in the functional selectivity towards ROCK and its isoforms. SB77 is more selective for ROCK1 than ROCK2 whereas Y27632 is non-isoform selective. Y27632 also inhibits other kinases such as PKC, PKA and MLCK, whereas such information about interaction of SB77 with other kinases is not clearly studied^28^. This warrants further studies to probe the selectivity of SB77 with other kinases as have been done for Y27632. Therefore, investigating the subcellular localization of both isoforms in the TM and SC may be beneficial to dissect the functions of ROCK 1 and 2 isoforms in regulating AH homeostasis. Additional studies with Y27632 in HOCAS derived from Indian eyes could also help resolve this issue. The increase in OF following SB77 was associated with increased tracer distribution (Fig. 2). This agrees with the findings of Yang *et al*. with Y27632^26^. Thus, the SB77-induced OF increase might be due to cellular relaxation of the TM, mediated through inhibition of Rho kinase^23,25^.

Previous reports indicate that the inhibition of Rho kinase with Y27632 or its structural analogues in TM cells induce time- and dose-dependent reversible changes in cell shape in association with loss of actin stress fibers, focal adhesions and cell-cell interactions^8,24,29^. Therefore, in the present study, we investigated how SB77 treatment altered cell morphology, actin stress fibers, cytoskeletal proteins, focal adhesions and ECM in HTM cells to gain insight into the molecular mechanism responsible for it’s OF enhancing property. SB77 induced alterations in cell shape leading to stellate appearance (S. Fig. 1), and reduction in actin stress fibers, cytoskeletal proteins and ECM proteins (Fig. 4). The observed changes were more noticeable in SB77 treated cells as compared to Y27632-treated cells, indicating that SB77 may be more potent in modifying the fibrotic disease processes in the TM that are associated with elevated IOP. The superiority of Y27632 in affecting OF relative to SB77 could be due to the difference in eye preparations. Y27632 may be more effective in enhancing OF in whole-eye perfusions than in the isolated anterior segment preparations but TM cell culture results in the present study indicate that Y27632 was less effective in affecting cell shape, actin cytoskeleton and ECM. This warrants further study to investigate the efficacy of Y27632 in isolated human anterior segment (HOCAS) preparations.

Our study revealed that the constitutive expression of Rho GTPase (active RhoA) was significantly reduced by both Y27632 (p = 0.029) and SB77 (p = 0.016) to essentially the same extent at the studied concentrations (Fig. 5). The decrease in active RhoA was associated with a marked reduction of myosin light chain (MLC) phosphorylation. MLC phosphorylation status is an indicator of cellular contraction, formation of actin stress fibers and focal adhesions^23,30^. Rho kinase mediates smooth muscle contraction by phosphorylating the myosin light chain directly and by decreasing myosin phosphatase activity indirectly, resulting in increased actin stress fibre formation^31^. In the present study, a markedly greater reduction in p-MLC was observed in HTM cells treated with SB77 as compared to Y27632 treated cells (Fig. 6C). This could be because SB77-induced changes in cell morphology, alterations in cytoskeleton and cell stiffness is mediated at least in part through the ROCK/MLC II pathway whereas the action of Y27632 is mediated through the ROCK/LIM kinase II/cofilin pathway. However, further studies are required to reveal SB77’s action through the ROCK/LIM kinase II/cofilin pathway.

Activation of Rho GTPase in TM cells increases cellular contraction and induces expression of genes related to αSMA, TGFβ2, CTGF, IL-1 and various ECM proteins in a Rho kinase-dependent manner^5,32^. Increase in ECM proteins activates Rho GTPase signalling and contractions in TM cells through a feed forward loop mechanism^7^. Inhibition of such a pathway would be expected to reduce cell stiffness and ECM fibrosis, and hence enhance OF. Since, the activation of Rho GTPase enhances TM cell stiffness and fibrogenic activity^33^, the effect of SB77 on fibrotic markers such as FSP-1, α-SMA and COL1A1, β-catenin and ECM (COL4A1 and fibronectin) proteins was investigated. We found that the three myofibroblast markers and fibronectin were significantly reduced by both Y27632 and SB77. A highly significant reduction of β-catenin expression was achieved with SB77 treatment as compared to control (p = 0.000); however, it was not found to be statistically significant when compared with Y27632 (Fig. 6; p = 0.088). In contrast, the expression of COL4A1 was significantly reduced by Y27632 (p = 0.001). This indicates that, like other ROCK inhibitors including clinically approved drugs such as ripasudil and netarsudil, SB77 may be a potential anti-fibrotic agent^34,35^. A detailed study related to its anti-fibrotic activity in comparison to ripasudil and netarsudil is warranted.

The clinically proven netarsudil not only showed high selectivity towards ROCK isoforms but also has inhibitory activity against the norepinephrine transporter^35^. RKIs are reported to interact with other kinases at high concentration including PKC^36^. PKC-dependent activation of α1-adrenergic receptor may have a role in regulating aqueous humor production. In addition, the permeability of SC can also be altered through the interaction with PKC (action similar to ripasudil). Therefore exploring other potential targets of SB77, such as inhibitory activity on β-adrenergic receptors and carbonic anhydrase, and promoting the uveoscleral outflow pathway by relaxing the ciliary muscle, may help to identify SB77 as a promising clinical candidate for the management of glaucoma.

**Figure 7:**
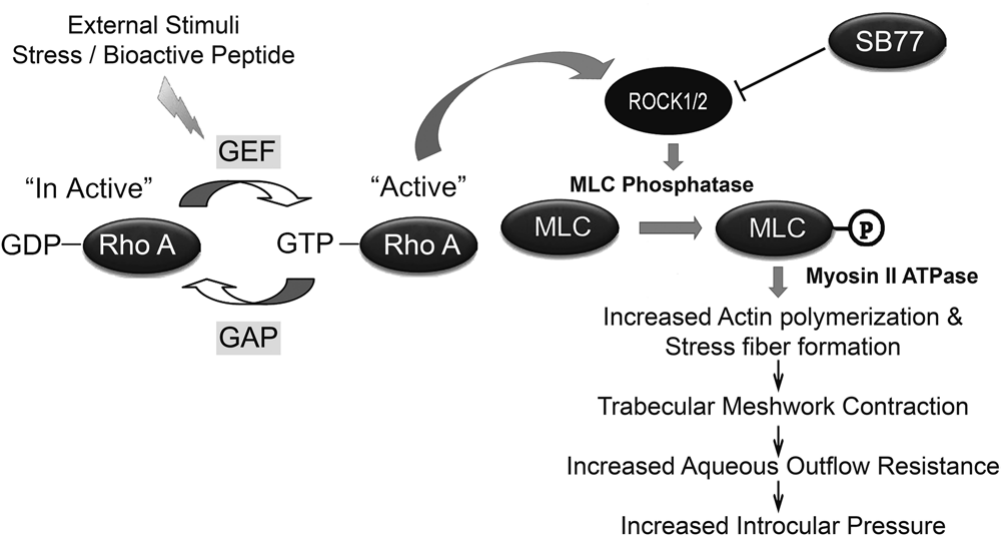
Schematic illustration of the effect of SB77 on IOP in HOCAS. Rho kinase is reported to mediate smooth muscle contraction by phosphorylating the myosin-light chain (MLC) directly and by decreasing myosin phosphatase activity indirectly, resulting in increased actin fiber formation and high intraocular pressure. SB77, a new Rho kinase inhibitor enhanced outflow facility in human eyes. This could be due to cellular relaxation of the TM mediated through inhibition of Rho kinase. This results in decreased p-MLC, altered cytoskeletal rearrangement and reduced fibrotic markers.

In conclusion, treatment with SB77 enhances aqueous OF with no sign of toxicity in HOCAS. The SB77-mediated OF increase is accompanied by the inactivation of RhoA and decreased p-MLC in cultured TM cells, and thus the action of SB77 is mediated at least in part through the ROCK/MLC II pathway (Fig. 7). TM cell culture studies revealed that SB77 was more effective in altering the cytoskeleton and reducing fibrotic markers than Y27632. Hence, SB77 may be a potential candidate for the management of glaucoma.

## Methods

### Human Donor Eyes

Fresh human donor eyes not suitable for corneal transplantation (based on insufficient corneal endothelial cell count) were obtained from the Rotary Aravind International Eye Bank, Aravind Eye Hospital, Madurai, India. They were handled in accordance with the Declaration of Helsinki. The study protocol was approved by the Institutional Review Board of the Aravind Medical Research Foundation, Madurai (ID NO. RES2013065BAS). The written consent of the donor or next of kin was also obtained. Donor eyes were enucleated within 4 h of death (mean elapsed time between death and enucleation was 2.9 ± 1.2 h) and kept at 4 °C until culture. Paired donor eyes were used for all HOCAS experiments. All eyes were examined under a dissecting microscope for any gross ocular pathological changes and only eyes without such changes were used. The presence or absence of glaucomatous changes in the study eyes were confirmed by histo-pathological analysis of the posterior segments as described earlier by our group (data not shown)^37^.

### Human Trabecular Meshwork (HTM) Cell Culture

Primary HTM cells were cultured from human donor eyes obtained from the eye bank. The TM was excised as ring of tissue and the HTM cell culture was established by extracellular matrix digestion method as described previously^4^. HTM cells were cultured on Corning cell culture flasks (Sigma Aldrich, MO, USA). The growth medium included Dulbecco’s modified Eagle’s medium (DMEM, Low glucose), 15% fetal bovine serum, 5 ng/ml basic fibroblast growth factor and antibiotics. Cells were maintained in 5% CO_2_ at 37 °C until 70–80% confluence. HTM cells from passages 3–6 were used for all experiments except outflow facility measurement.

### HOCAS Setup

HOCAS was established using paired human donor eyes as described previously^38^. Briefly, the anterior segments were dissected out after removing vitreous, lens and iris leaving the ciliary body from the donor eyes. They were mounted onto a specially designed Petri dish containing Dulbecco’s Modified Eagle’s Medium (DMEM, containing 4500 mg glucose/L, L-glutamine, NaHCO_3_ and pyridoxine HCl, Sigma-Aldrich, MO, USA) supplemented with gentamycin (15 mg/L, Sigma-Aldrich, MO,USA) and antibiotic/antimycotic solution (penicillin G, 100 U/ml; streptomycin sulphate, 100 μg/ml; amphotericin B, 0.25 μg/ml: Sigma-Aldrich, MO, USA)^27^. Topical antibiotic solution was applied to the cornea. Stopcocks were closed and the eye segments were transferred to an incubator (37 °C, 5% CO_2_) and attached to pressure transducers (APT 300 pressure transducers, Harvard Apparatus, MA, USA). The pump (PHD 2000 Ultra Syringe Infusion only pump, Harvard Apparatus, MA, USA) was started; infusion set at 3 μl/minute and pressure was monitored on a computer connected to the Power Lab system with Lab Chart Pro software (AD Instruments, CO, USA). The eyes that maintained IOP within physiological range (10–21 mm Hg) for ~2 days were subsequently used for drug treatment.

### Drug Treatment

After baseline (BL) equilibration (~2 days), anterior segments were exchanged with 5 ml of SB77 (0.1, 10 or 50 µM) at 200 µl/minute while the IOP was maintained at 8 mmHg. Then the spontaneous pressure was re-established by continued perfusion at 3 µl/minute with the medium containing the test drug. The contralateral anterior segment received the medium with vehicle (dmethylsulfoxide (DMSO): 0.001–0.5% v/v). AH outflow facility (OF) (µl per minute/mm Hg) was calculated as the ratio between the inflow rate (µl/minute) and the measured IOP (mm Hg). OF was calculated every hour as the average of 6 values recorded every 10 minutes, beginning 3 h before the drug infusion and continuing for the duration of the culture. The effect of drug on OF was calculated after 3, 12 and 24 h and expressed as the percentage change from vehicle control after correction for the corresponding treated and control baselines (Percentage change in OF = [(Treated/BL/Control/BL) −1] X100). The average OF value between 3–4 h before drug infusion was taken as baseline. At the conclusion of the studies, anterior segments were fixed by perfusion with 7 ml of 4% paraformaldehyde (at 8 mm Hg) for assessing the morphology of aqueous outflow tissues. In addition, the hydrodynamic pattern after drug treatment was visualized using red fluorescent microspheres (see below).

### Hydrodynamic Pattern after SB77 Treatment

In order to visualize the flow pattern following drug treatment, the anterior chamber contents of the eyes were exchanged with Dulbecco’s PBS (DPBS) containing red fluorescent microspheres (0.2 µm, 0.002% v/v; 7 ml) (Thermo Fisher Scientific, Waltham, MA; 0.2 µm, Ex. 580 nm; Em. 605 nm) at 200 μl/minute (at 8 mmHg) as described previously^26^ and then the flow rate was resumed at 3 µl/minute for 1 h. Eyes were then fixed by perfusion at 8 mmHg with Karnovsky’s fixative (2.5% glutaraldehyde and 2% paraformaldehyde, pH 7.3). Anterior segments were immersed overnight at 4 °C in the same fixative for further processing. All fluorescent microsphere-treated fixed eyes were cut into sixteen radial quadrants and each quadrant was processed by the method described previously^26^. Briefly, each quadrant was cut into frontal sections, along a plane tangential to the corneo-scleral limbus and perpendicular to the ocular surface. Then the cell nuclei in each section were stained with DAPI (Thermo Fisher Scientific, Waltham, MA) followed by three washes with PBS. The sections were mounted and examined using the Leica TCS-SP8 confocal microscope (Leica, Heidelberg, Germany). Fluorescent microspheres were visualized with a 20X objective and the Z-stack images were reconstructed as a 2D-average projection along a fixed axis. Images were taken from all sections containing SC in order to evaluate the distribution of tracer along the inner wall of SC and TM. The thickness of the TM (the perpendicular distance from the innermost uveo-scleral beams to the inner wall of SC) containing red fluorescence was calculated using Image J software. The confocal images with whole meshwork and clearly visible SC were selected for measurement and those confocal images were not necessarily from paired eyes because of their non-suitability for image acquisition. A minimum of 5 frontal sections per eye were analysed (n = 3–7 images per eye). The TM thickness from the calibrated images was measured at three different locations (at every 200 µm length), the average thickness of TM was then calculated and analysed (S. Fig. 1).

### Cytotoxicity Assay

The effect of SB77 on cell viability of HTM was measured by MTT based assay as per the manufacturer’s protocol. Briefly, 10^4^ cells per well were seeded into a 96-well plate and cultured until 80–90% confluent. Cells were serum starved for 12 h and fed with fresh media with or without SB77 (0.1–100 μM) for 24 h. After 24 h, the cells were treated with 5 mg/mL MTT (Sigma Aldrich, MO, USA) for 4 h at 37 °C. The MTT solution was aspirated and the formazan crystals were dissolved in DMSO and the optical absorbance for formazan was measured at 570 nm in an automatic plate reader (Spectramax M3, Molecular devices, PA, USA), with a reference wavelength of 655 nm. Experiments were conducted in triplicate.

### TUNEL Assay

The effect of SB77 on apoptosis of HTM cells was determined using TUNEL Apoptosis Detection kit, (Trevigen, WI, USA) according to the manufacturer’s protocol. Briefly, 10^4^ HTM cells were plated on glass coverslips in 6-well plates and grown at 37 °C for 24 h. The cells were treated with either DMSO as vehicle control or 50 μM drug (SB77 or Y27632)-containing medium for an additional 24 h. At the end of drug treatment, the cells were fixed with 4% paraformaldehyde in PBS for 15 minutes, washed and permeabilized with Proteinase K for 5 minutes. TUNEL labelling was performed at 37 °C for 60 minutes and the reaction was stopped using stop buffer followed by PBS washing. The slides were incubated with Strep-Fluor solution for 20 minutes at room temperature in dark, mounted with vecta-shield mounting medium containing DAPI (Vector Laboratories, Burlingame, CA) and observed under a fluorescence microscope (AXIO Scope A1, Zeiss, Germany). Cells treated with 1 unit/mL DNase I for 10 minutes served as a positive control. The number of apoptotic cells was determined by counting TUNEL-positive cells on fluorescent microscopic images under 10X objectives.

### RhoA Activation Assay

The effect of SB77 on RhoA activation in cell lysates of HTM after treatment with SB77 or Y27632 was evaluated by a pull-down assay using the RhoA Activation Assay Biochem kit (B124, Cytoskeleton, CO, USA) according to the manufacturer’s protocol. Briefly, equal amounts of protein from each of the treatments were incubated with Rhotekin-Rho binding domain-agarose beads using gentle rocking at 4 °C for 1 h. The agarose beads were then washed thrice and suspended in 2X Laemmli sample buffer. The GTP-bound form of RhoA (Active form) was detected by Western blot analysis using anti-RhoA specific monoclonal antibody. Data were normalized to total RhoA as loading control.

### Immunofluorescence Analysis

HTM (1 × 10^3^) cells were plated on glass coverslips and cultured until 80% confluence. After respective drug treatment for 2 h, the cells were fixed with 4% paraformaldehyde and permeabilized with 0.5% Triton X-100 for 5 minutes. The samples were blocked with avidin-biotin blocking system for 10 minutes. The cells on coverslips were incubated overnight with anti-vinculin (1:200), anti-collagen 1 A (1:500), anti-fibronectin (1:200), anti-pMLC, (1:200) or anti-vimentin antibody (1:200) diluted in 1% BSA at 4 °C. Coverslips were washed thrice with PBS and incubated with FITC- conjugated secondary antibody for 1 h. Cells without primary antibody served as a negative control. In order to stain actin stress fibers, the permeabilized cells were treated with FITC-phalloidin for 1 h. After washing, the stained cells were mounted with anti-fade mounting media containing DAPI and observed under a fluorescence microscope (AXIO Scope A1).

### Quantitative Real-Time PCR

Activation of RhoA results in trans-differentiation of HTM cells into myofibroblasts and induces a fibrogenic response^33^. Therefore, in the present study, the mRNA expression profiling of three well recognized myofibroblast markers (fibroblast specific protein-1 (FSP-1), α-SMA and COL1A1), β-catenin (cell-cell adhesion protein) and ECM proteins (COL4A1 and fibronectin) in cultured HTM cells after treatment with SB77 or Y-27632 were studied by quantitative real time-PCR. Briefly, Total RNA extracted from HTM cells using the RNeasy Mini Kit (Qiagen, CA, USA) was quantitated using a NanoDrop 2000 UV-Vis Spectrophotometer (Thermo Scientific, DE, USA). Equal amounts of RNA were then reverse transcribed using SuperScript III reverse transcriptase (Invitrogen, CA, USA) according to manufacturer’s protocol. Quantitative PCR was performed using a 7900 HT Real-Time PCR system (Applied Bio systems). Reactions were performed in 20 µl of reaction mixture containing 10 µl PCR master mix (QuantiTect SYBR Green PCR kit, Qiagen, CA, USA), 2 μl of cDNA and 0.5 μM of primer pairs (S. Table 1). The thermal cyclic conditions followed were: 95 °C for 7 minutes followed by 45 cycles of 95 °C for 15 seconds and 60 °C for 30 seconds. All PCR reactions were carried out in triplicate. The specificity of primers was validated by a dissociation curve analysis and the fold change in expression of each gene was calculated using the 2^−ΔΔCt^ method, with GAPDH as an internal control.

### Immunoblot Analysis

Total protein cell lysates were prepared from confluent cultures of HTM cells after treatment with 50 μM of SB77 or Y-27632. Total protein in the cell lysate was determined by Bicinchoninic acid assay kit (Bio-Rad, Hercules, CA). Samples containing equal amount of protein were mixed with Laemmli buffer and separated by 10% SDS-PAGE, followed by transfer of resolved proteins onto nitrocellulose membranes. Membranes were blocked for 1 h in Tris-buffered saline containing 0.1% Tween 20 and 5% (w/v) non-fat dry milk at room temperature, and subsequently probed with primary antibodies (anti-vinculin, anti-fibronectin, and anti-vimentin (Santa Cruz Biotechnology, Dallas, TX, USA) and anti-Collagen-1A (Abcam, Cambridgeshire, UK)) in conjunction with horseradish peroxidase-conjugated secondary antibodies. Detection of immunoreactivity was performed by enhanced chemiluminescence Western blotting substrate (Pierce; Thermo Fisher Scientific, USA). Densitometry on immunoblot films was performed using NIH Image J software (http://imagei.nih.gov.ij/) provided in the public domain by NIH, Bethesda, MD, USA). Data were normalized to the loading control (anti-β–actin). Fold change in expression was calculated after normalization with control.

### Statistical analysis

Statistical analysis was carried out using Graph Pad Prism (Graph Pad 7.0, USA). Data are presented as mean ± SD values unless otherwise mentioned. For OF studies, a paired-t-test was used to compare the significance of difference between drug-treated and vehicle control eyes. A 2-sample un-paired t-test was used to compare the significance of difference between drug-treated and vehicle control eyes in non-paired TM thickness measurement studies. For immunoblot and quantitative RT PCR, the significance in fold change between treated and controls were calculated by paired Student’s t-test.

## Electronic supplementary material

Supplementary Dataset 1

## Data Availability

The datasets generated during and/or analyzed during the current study are available from the corresponding author on reasonable request.
