# Supplementary material for: SB772077B, A New Rho Kinase Inhibitor Enhances Aqueous Humour Outflow Facility in Human Eyes
Source: Sci Rep. 2018 Oct 19;8:15472. doi: 10.1038/s41598-018-33932-8 (PMC6195566; doi:10.1038/s41598-018-33932-8)
Supplement: Supplementary file 1 — Supplementary Dataset 1 [file 41598_2018_33932_MOESM1_ESM.pdf]

Supplementary Information

SB772077B, A NEW RHO KINASE INHIBITOR ENHANCES AQUEOUS HUMOUR OUTFLOW FACILITY IN HUMAN EYES

Soundararajan Ashwin Balaji [1], Srinivasan Senthilkumari * [1], Chidambaranathan Gowri Priya[2], Subbaiah Krishnadas [3], B'Ann T.Gabelt [4], Paul L. Kaufman[4], Veerappan Muthukkaruppan[5]

[1]Department of Ocular Pharmacology, Aravind Medical Research Foundation, #1, Anna Nagar, Madurai-20, Tamilnadu, INDIA E-mail: ss_kumari@aravind.org; aashwinbtech@gmail.com

[2] Department of Immunology & Stem Cell Biology, Aravind Medical Research Foundation, #1, Anna Nagar, Madurai-20, Tamilnadu, INDIA . E. mail: gowri@aravind.org

[3] Glaucoma Clinic, Aravind Eye Hospital, #1, Anna Nagar, Madurai-20, Tamilnadu, INDIA. E-mail: krishnadas@aravind.org

[4] Department of Ophthalmology & Visual Sciences, University of Wisconsin, Madison, Wisconsin, USA. E-mail: btgabelt@wisc.edu; paul.kaufman@wisc.edu

[5] Advisor, Aravind Medical Research Foundation, #1, Anna Nagar, Madurai-20, Tamilnadu, INDIA. E-mail: muthu@aravind.org

* **Corresponding Author:**

Srinivasan Senthilkumari, M. Pharm, Ph. D

Department of Ocular Pharmacology, Aravind Medical Research Foundation

#1, Anna Nagar, Madurai-625020, Tamilnadu, India

Tele (0): +91-452-4356550; extn. 438; Fax: +91-452-2530984; e.mail: ss_kumari@aravind.org

Supplementary Figures

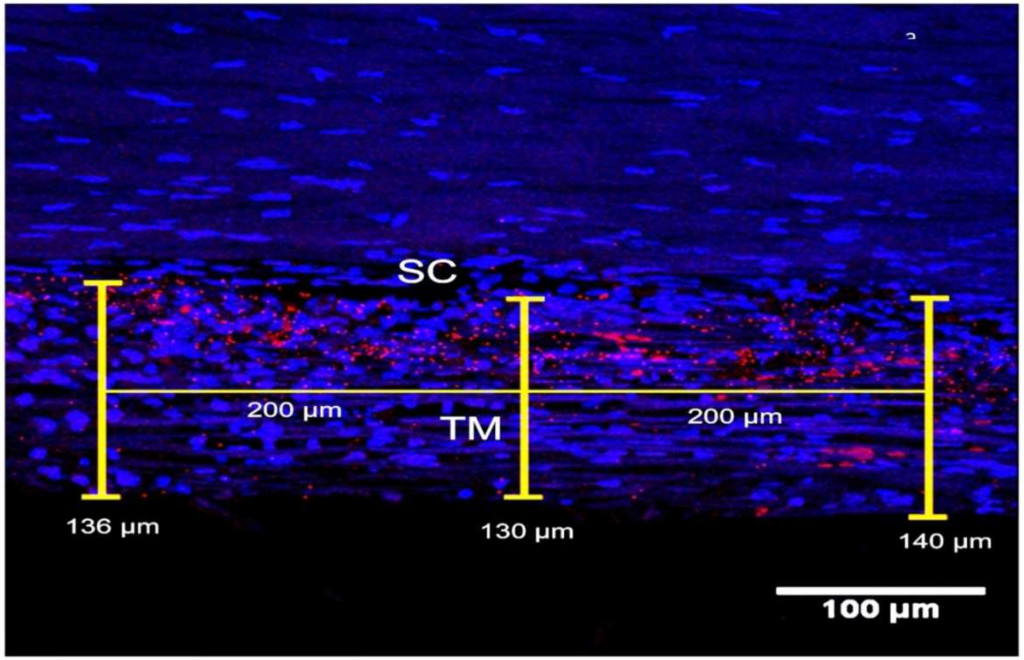

S. Fig 1: Measurement of TM thickness in red tracer-decorated region of the anterior segment.

Detailed procedure is given in method section. The TM thickness from the calibrated images was measured at three different locations (at every 200 µm length), and the average thickness of TM was then calculated and analyzed. Cell nuclei were stained with DAPI (blue).

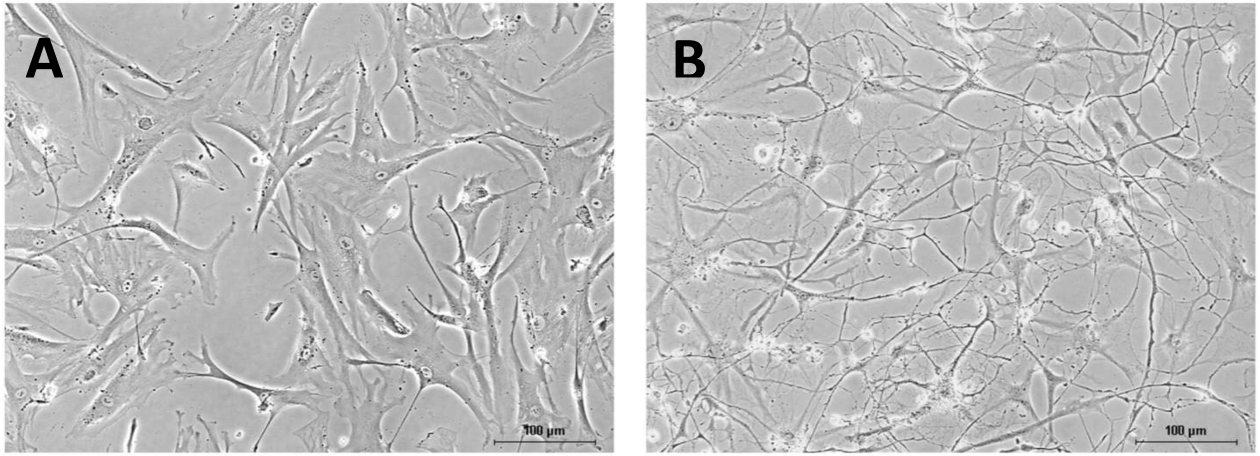

S. Fig 2: Effect of SB77 on cell morphology. HTM cells after treatment with DMSO as vehicle control (A) and 50µM SB77 (B) for 24 h are shown. HTM cells after treatment with SB77 showed retraction of cells (arrow) that led to stellate appearance as compared to DMSO treated cells.

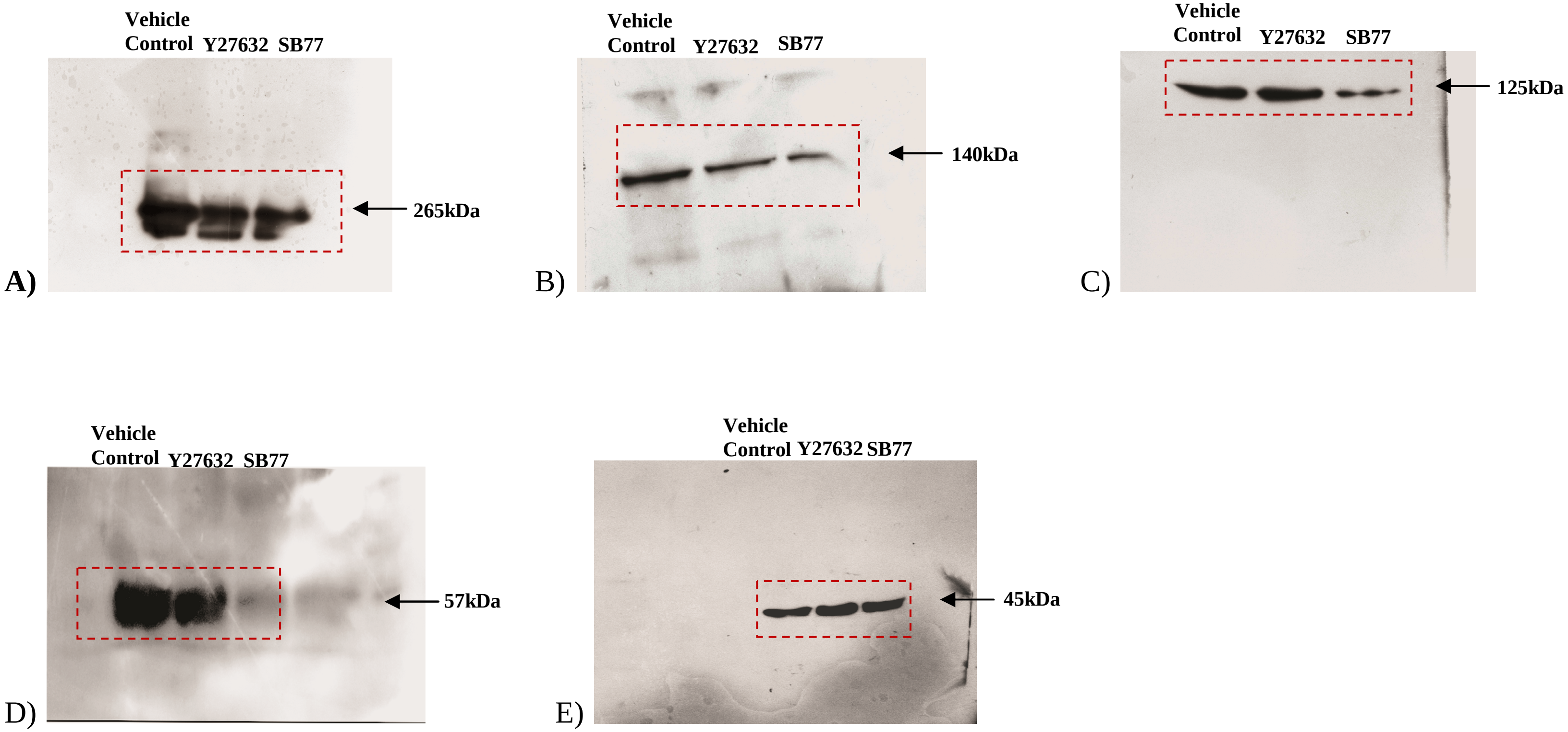

S. Fig 3: Full length blots of Fig.4: Immunoblot analysis showing the effect of SB77 on the expression of focal adhesion and ECM proteins, Fibronectin (A), Collagen IA (B), Vinculin (C), Vimentin (D) and β-Actin (E). Red-dotted line indicates the cropped location of the blots.

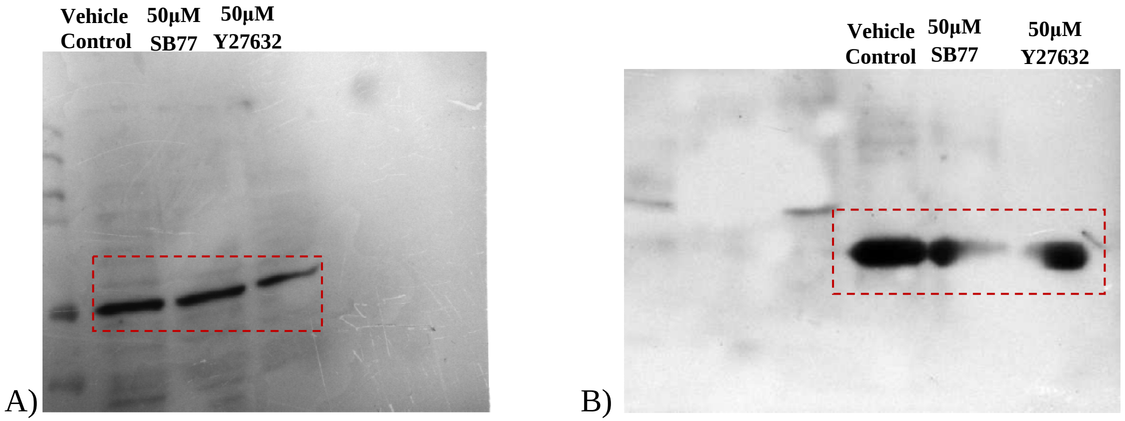

S.Fig 4: Full length blots of Fig.5. Immunoblot analysis of Total-RhoA (A) and GTP-RhoA (B) were shown. The amount of reduction in activated RhoA was determined by a pull-down assay. Detailed information is given in methods. Red-dotted line indicates the cropped location of the blots.
